# Engineered Human Intervertebral Disc Model Inducing Degenerative Microglial Proinflammation

**DOI:** 10.3390/ijms232012216

**Published:** 2022-10-13

**Authors:** Min-Ho Hwang, You Jung Kang, Hyeong-Guk Son, Hansang Cho, Hyuk Choi

**Affiliations:** 1 Department of Medical Sciences, Graduate School of Medicine, Korea University, Seoul 08308, Korea; 2 Institute of Quantum Biophysics, Sungkyunkwan University, Suwon 16419, Korea; 3 Department of Biophysics, Sungkyunkwan University, Suwon 16419, Korea; 4 Department of Intelligent Precision Healthcare Convergence, Sungkyunkwan University, Suwon 16419, Korea

**Keywords:** intervertebral disc degeneration, human nucleus pulposus, microglia, proinflammation, chemotaxis

## Abstract

Degeneration of the intervertebral disc (IVD) is a major contributor to low back pain (LBP). IVD degeneration is characterized by abnormal production of inflammatory cytokines secreted by IVD cells. Although the underlying molecular mechanisms of LBP have not been elucidated, increasing evidence suggests that LBP is associated particularly with microglia in IVD tissues and the peridiscal space, aggravating the cascade of degenerative events. In this study, we implemented our microfluidic chemotaxis platform to investigate microglial inflammation in response to our reconstituted degenerative IVD models. The IVD models were constructed by stimulating human nucleus pulposus (NP) cells with interleukin-1β and producing interleukin-6 (129.93 folds), interleukin-8 (18.31 folds), C-C motif chemokine ligand-2 (CCL-2) (6.12 folds), and CCL-5 (5.68 folds). We measured microglial chemotaxis (*p* < 0.05) toward the conditioned media of the IVD models. In addition, we observed considerable activation of neurodegenerative and deactivation of protective microglia via upregulated expression of CD11b (*p* < 0.001) and down-regulation of CD206 protein (*p* < 0.001) by soluble factors from IVD models. This, in turn, enhances the inflammatory milieu in IVD tissues, causing matrix degradation and cellular damage. Our findings indicate that degenerative IVD may induce degenerative microglial proinflammation, leading to LBP development.

## 1. Introduction

Low back pain (LBP) has a lifetime prevalence of 60–80% among the general population [1]. Despite the enormous financial burden of LBP and the demand, it places on the spinal healthcare system, its underlying pathological mechanisms remain unknown. Although the underlying mechanism and major causes of LBP have not been completely elucidated, increasing evidence suggests that degeneration of the intervertebral disc (IVD) is strongly linked to LBP [1,2].

The IVD is a soft tissue between the vertebrae that absorbs and distributes applied loads, making the spine flexible. The IVD is a fibrocartilaginous structure that is composed of two major distinct components: central nucleus pulposus (NP), surrounded by peripheral annulus fibrosus (AF). Cartilaginous NP tissue is rich in proteoglycans and water, which enables the IVD to resist compressive forces. Collagenous AF stabilizes tensile forces through its collagen lamellar structure [3]. Under healthy conditions, IVD tissue is avascular and aneural, except for the outer third of the AF. In contrast, vascular structures have been observed in the deeper IVD region in degenerative conditions with LBP [1,4,5]. The structural deficits that occur during IVD degeneration, including tears and clefts in IVD tissue by immoderate biomechanical loading, permit the infiltration and activation of immune cells, including leukocytes, monocytes, macrophages, and neuroglial cells, such as microglia in the spinal cord [2,6,7,8]. The infiltrated immune cells secrete pro-inflammatory cytokines, including IL-1β. IL-1β can bind to IL-1R1 (IL-1 receptor type 1) and the IL-1β/IL-1R1 complex resulting in the activation of important signalling proteins, such as NF-κB (p65 and p50 subunits), which control the expression of a number of inflammatory and catabolic genes in human IVD cells. Consequently, IVD cells stimulated by this pro-inflammatory cytokine express several inflammatory mediators and catabolic enzymes such as IL-6, IL-8, and CCLs (C-C motif chemokine ligands) [2,9,10]. These factors can cause ECM components to continue to degrade and generate inflammation, which can result in the creation of physical space and/or a physiological reaction that allows vascular structures to penetrate deeper into the NP region. Invasive vascular structures trigger a cascade of new propagative IVD degeneration events, such as an inflammatory response by interactions between NP and immune cells [11,12,13]. Furthermore, although the molecular mechanism of IVD degeneration is still poorly understood, multiple lines of evidence suggest that excessive and/or abnormal production of inflammatory cytokines, chemokines, and catabolic matrix enzymes in IVD tissues during these interactions could be the specific end-product of IVD degeneration that eventually causes LBP [2,6,7,11,12,13]. Additionally, evidence suggests that these factors induce the activation and infiltration of neuroglial cells, such as microglia in the spinal dorsal horn, which are responsible for neuroinflammation during IVD degeneration and the onset of LBP [14,15,16]. Therefore, the understanding of microglial functions in IVD degeneration with LBP would contribute to the identification of promising targets for IVD degeneration-mediated neuroinflammation and the development of novel therapeutic strategies in advance.

Herein, we studied the effects of the inflammatory microenvironment and potential contributing factors of human NP cells on microglial phenotype conversion and chemotaxis. IL-1β-mediated inflammation is a major degenerative source in the pathomechanism of IVD degeneration, which in turn leads to microenvironmental and phenotypic changes in IVD cells/tissues. We used the conditioned medium of human NP cells with IL-1β stimulation as an in vitro degenerative IVD model. Moreover, using a previously developed microfluidic chemotaxis platform [17], we studied the motility and/or activation of microglia in response to potential contributing factors from degenerative NP cells because the infiltration of microglia and interaction between NP and microglia is a primary cause of the exacerbating neuroinflammation in degenerative IVD [14,15,16].

## 2. Results

### 2.1. IL-1β Stimulation Induces the Inflammatory Response by Modulating Gene and Protein Expression of Inflammatory Mediators and by Nucleus Translocation of NF-κB p65 Protein in Human NP Cells

**Figure 1:**
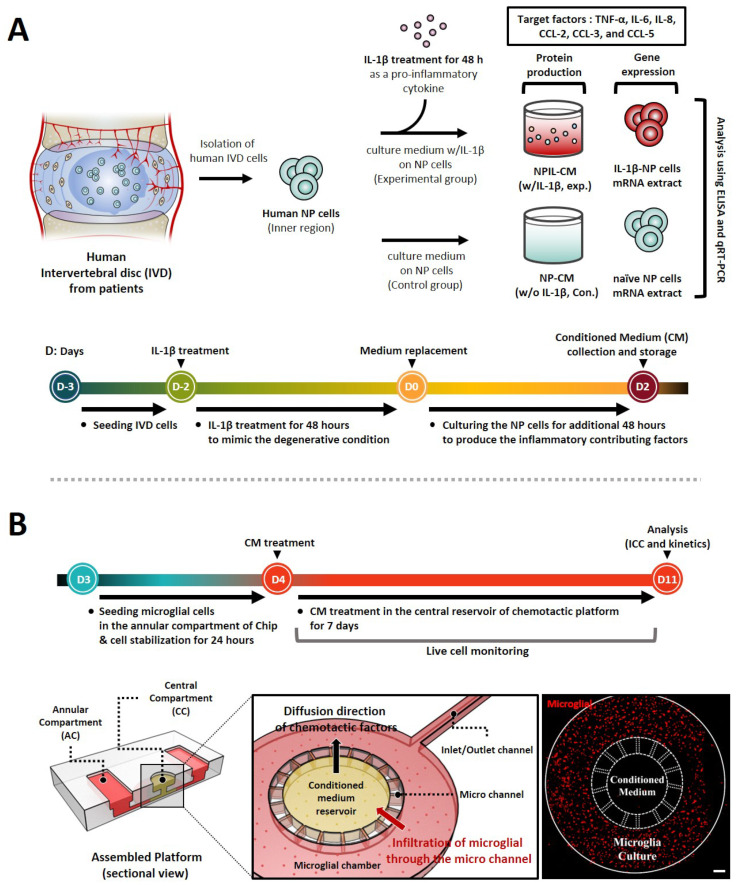
Schematic of stepwise experiment set-up and timeline for replication of interactions between human NP cells and microglia using a microfluidic chemotaxis platform. (**A**) Following initial injury, human NP cells are stimulated by pro-inflammatory cytokines IL-1β, inducing the secretion of diverse inflammatory mediators and chemokines. Thus, we evaluated the protein production and gene expression of these factors, including TNF-α, IL-6, IL-8, and CCLs. (**B**) In the second phase, the degenerative microenvironment of human NP cells can permit the infiltration and activation of immune cells, such as microglia, which are believed to be responsible for neuro-inflammation. Thus, we assessed the microglia’s chemotaxis and its activation using a microfluidic chemotaxis platform. The platform is composed of two major units, including a central compartment (CC) and an annular compartment (AC), connected by thin microchannels. The fluorescence image shows the experiment set-up on the platform for the microglia chemotaxis study. Scale bar, 500 μm.

**Figure 2:**
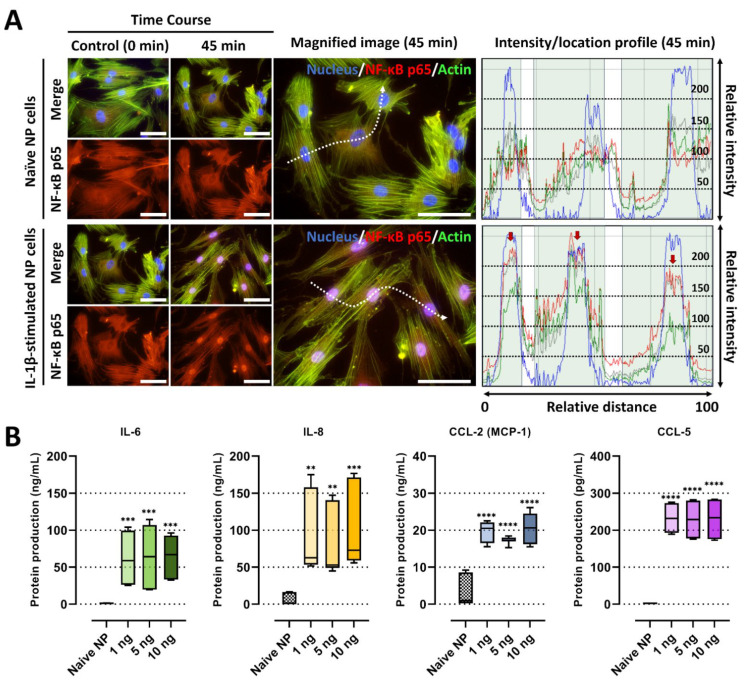
IL-1β stimulation on human NP cells for degenerative IVD conditions. (**A**) Fluorescence image and quantification of the fluorescence intensity of NF-κB p65 protein in human NP stimulated by recombinant IL-1β 10 ng/mL. The red arrow indicates the preferential distribution of NF-κB p65 protein in the nucleus. Scale bars, 100 μm. (**B**) The protein production of IL-6, IL-8, CCL-2 and CCL-5 on human NP cells with/without IL-1β treatment in a dose-dependent manner. The values are reported as the mean ± standard error of four independent experiments. Scale bars, 225 μm. ** *p* < 0.01, *** *p* < 0.001, and **** *p* < 0.0001 as compared with naïve NP cells.

IL-1β plays a major role in the development of IVD by modulating diverse inflammatory cytokines via the NF-κB pathway [2,10]. In this study, we developed in vitro IVD models by stimulating human NP cells with IL-1β, which initiated detrimental inflammation via NF-κB p65 translocation (Figure 1). We first validate our models by investigating the translocation of NF-κB p65 to the nucleus in the human NP cells in the presence of IL-1β (10 ng/mL). Quantitatively, p65 activity calculated from the average intensity value in IL-1β-stimulated NP cells exhibited an increase, and most of the detected intensity was located in the nucleus within 45 min, in contrast to naïve NP cells (Figure 2A).

**Figure 3:**
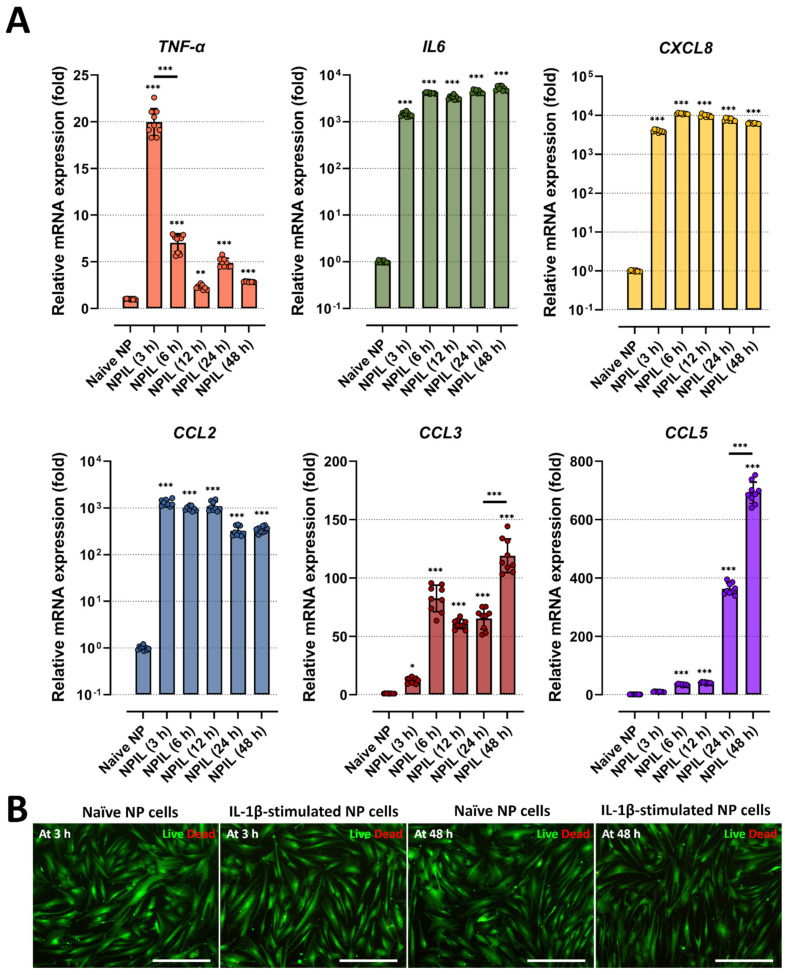
IL−1β stimulation induces the gene expression of inflammatory mediators and chemokines on human NP cells. (**A**) Comparison of gene expression for inflammatory cytokines and chemokines from human NP cells with/without IL−1β treatment in a time-dependent manner. (**B**) Assessment of cytotoxicity by using Live/Dead assay. The results showed that human NP cells treated with IL−1β did not exhibit any difference in cell viability. The values are reported as the mean ± standard error of four independent experiments. Scale bars, 225 μm. * *p* < 0.05, ** *p* < 0.01, and *** *p* < 0.001 as compared with naïve NP cells. The line indicates the comparison with each group.

Secondly, we assessed the levels of pro-inflammatory factors in the conditioned media of IL-1β-stimulated NP cells, which may contribute to cell-to-cell communication and chemotaxis, leading to detrimental inflammation. The data showed that IL-1β-stimulated NP cells secreted significantly higher levels of major chemotactic factors, including IL-8, CCL-2, and CCL-5, and pain-related factors, such as IL-6, than the naïve NP cells (Figure 2B). Furthermore, we tested the gene expression of pro-inflammatory cytokines as *TNF-α*, inflammatory mediator *IL6*, and chemokines as *CXCL8, CCLs* including *CCL2*, *3*, *5*, in human NP cells under 10 ng/mL of IL-1β stimulation. Our results show that the mRNA expression of all tested markers on human NP cells at 48 h was upregulated by the stimuli (Figure 3A).

Third, we examined whether the IL-1β-driven inflammation can damage cells. To this end, we stimulated NP cells with IL-1β stimulation at a dose of 10 ng/mL, which is the maximum dose used in this study, and measured cell viability by live/dead assay. Compared with naïve NP cells, human NP cells treated with IL-1β did not exhibit any difference in cell viability (Figure 3B).

Collectively, these results indicate that IL-β stimulation initiated the pro-inflammatory response of human NP cells by activating NF-κB p65 pathway and producing inflammatory mediators and chemotactic factors.

### 2.2. Significant Migration of Microglia towards Soluble Factors Derived from IL-1β-Stimulated NP Cells

We recently developed a chemotactic microfluidic platform to investigate the innate immune response toward pro-inflammatory mediators quantitatively. In this study, we employed our microfluidic platform to characterize the kinetics of microglial migration driven by potential contributing factors from NP cells. To this end, human adult microglia (SV40) were cultured in the annular chamber while we added NPCM or NPIL in the central chamber. For the control, we added the cell culture media in the central chamber. Afterward, we monitored individual microglial cells migrating to the central chamber with conditioned medium from NP cells with or without IL-1β stimulation for two days under the live cell microscopy. We observed that microglia were migrating towards the central chamber upon the addition of NPIL in 48 hrs while they were not migrating towards both naïve medium and NPCM.

**Figure 4:**
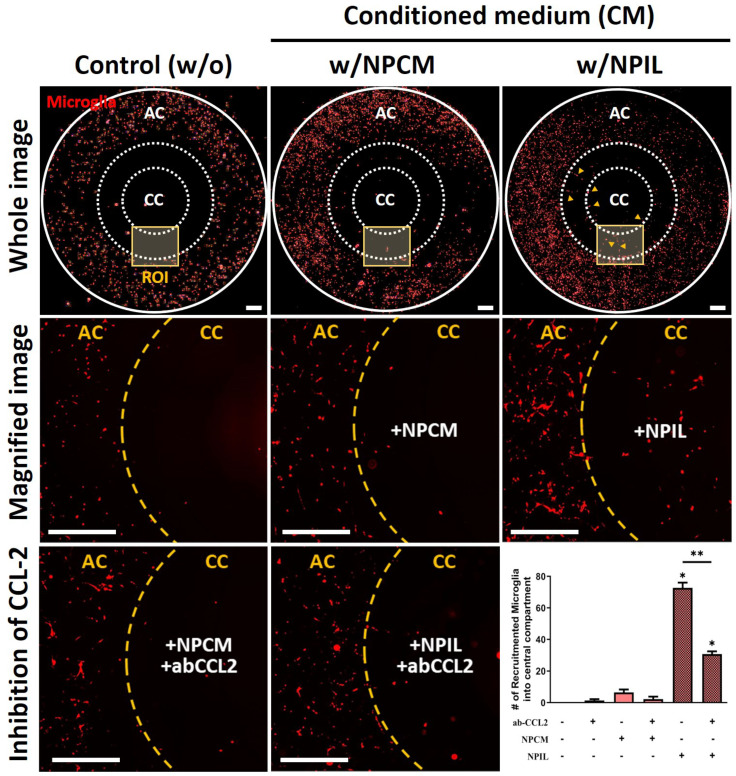
Recruitment of microglia using conditioned medium derived from IL−1β−stimulated human NP cells. Significant recruitment of microglia is observed in the central compartment (CC) containing NPIL (conditioned medium of IL−1β−stimulated NP cells) from the annular compartment (AC). Additionally, results show that neutralizing CCL−2 reduced the chemotaxis effects on microglia. Scale bars, 500 μm. *p < 0.05, **p < 0.01 as compared with naïve microglia cells. The line indicates the comparison with each group.

Among the chemokines in NPIL, CCL-2 is a well-known chemoattractant for innate immune cells, including microglia, which is found to be substantially higher levels in painful discs than in non-painful discs. We hypothesized that the chemokine in the NPIL may serve significant roles in the microglia migration. To test our hypothesis, we exposed microglia to either NPIL or NPIL supplemented with CCL-2 neutralizing antibodies. The results showed that NPIL with neutralizing CCL-2 significantly reduced microglial recruitment towards the central compartment compared with the NPIL alone group. The other groups did not exhibit any significant differences in migration (Figure 4). Collectively, these results indicate that CCL-2 in NPIL can be the major chemoattractant for microglial migration, which could initiate microglia-mediated neuroinflammation in advance.

### 2.3. Conditioned Medium from IL-1β-Stimulated NP Cells Induces Phenotypic Switching on Microglia

We next examined whether the pro-inflammatory IVD microenvironment could induce phenotypic switching in innate immune cells, such as microglia, which is known to play significant roles in neuroinflammation during IVD degeneration and in the onset of pain. To this end, we treated microglia with conditioned media of NP cells (NPCM) or that of IL-1β-stimulated NP cells (NPIL) and investigated the effects of conditioned medium from human NP cells on microglia for phenotypic switching. We particularly studied on the expressions of CD11b and CD 206, which are major markers for pro-inflammatory and anti-inflammatory phenotypes, respectively.

**Figure 5:**
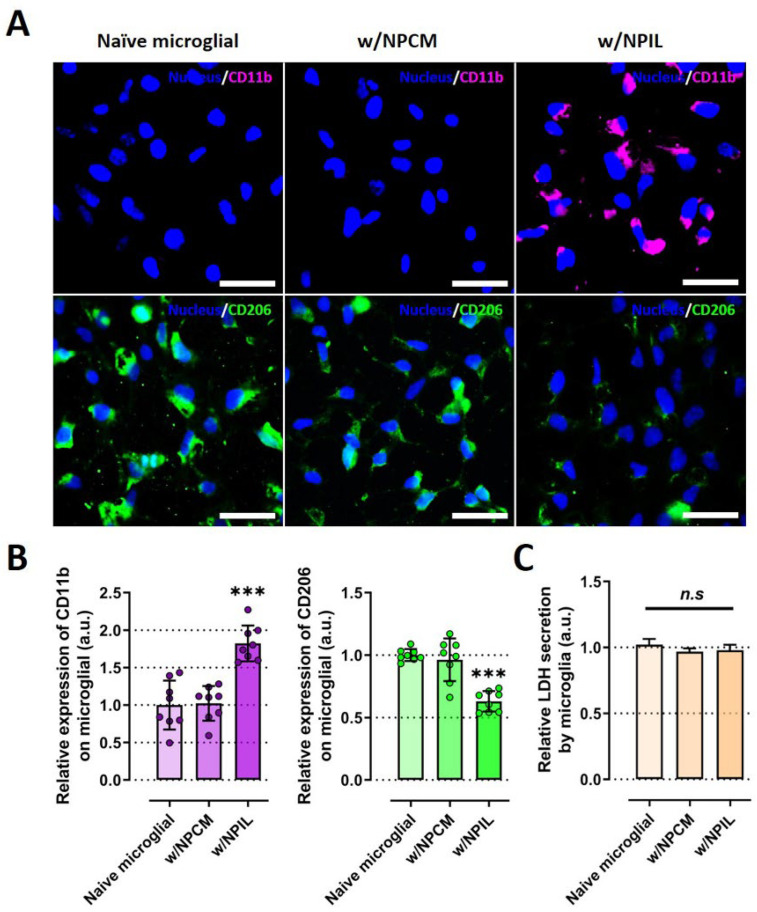
IL-1β-stimulated human NP cells induce a microglial phenotypic switch to proinflammation. (**A**) Immunofluorescence data revealed condensed CD11b or CD206 expression in the cytoplasm of microglia cultured in NPIL or NPCM compared with naïve microglia, respectively. (**B**) Quantification of the fluorescence intensity of the CD markers in microglia. The results showed the increased expression of CD11b protein (M1 macrophage maker), and the decreased expression of CD206 (M2 macrophage marker), in microglia under the presence of NPIL. (**C**) Assessment of microglial cytotoxicity with NPCM or NPIL by using LDH assay. Microglia cultured in NPCM or NPIL did not exhibit any difference in cell cytotoxicity compared with naive microglia. Scale bars, 50 μm. *** *p* < 0.001, and n.s., no significant difference, as compared with naïve microglia. The line indicates the comparison with each group. Each dot represents the mean of six samples in an independent experiment.

Fluorescence images and quantitative results revealed that CD11b protein in microglia was upregulated in the presence of NPIL, whereas CD206 protein was downregulated in the condition, compared with both naïve and NPCM-treated microglia (Figure 5A,B). Similar to the IL-1β experiment, because the treatment of NPCM or NPIL can damage the microglia, we assessed cell cytotoxicity using LDH assay. neither NPCM nor NPIL exhibited significant toxicity to microglia. Microglia cultured in NPCM or NPIL did not exhibit any difference in cell cytotoxicity compared with naive microglia (Figure 5C).

These results demonstrate that potential contributing factors derived from IL-1β-stimulated NP cells could induce phenotypic changes in microglia into M1 immune cells, which are thought to induce inflammation in the IVD.

## 3. Discussion

Neuroinflammation is thought to play a pivotal role in the development of chronic LBP during IVD degeneration [14,15,16,18,19]. During IVD degeneration, an inflammatory microenvironment is established within the NP tissues of the IVD and in the peridiscal space [15]. The aim of this study was to confirm the effects of this microenvironment on microglia, which is believed to be responsible for neuroinflammation. The major role of microglia with regard to NP tissues in exacerbating IVD degeneration is still not well understood.

**Figure 6:**
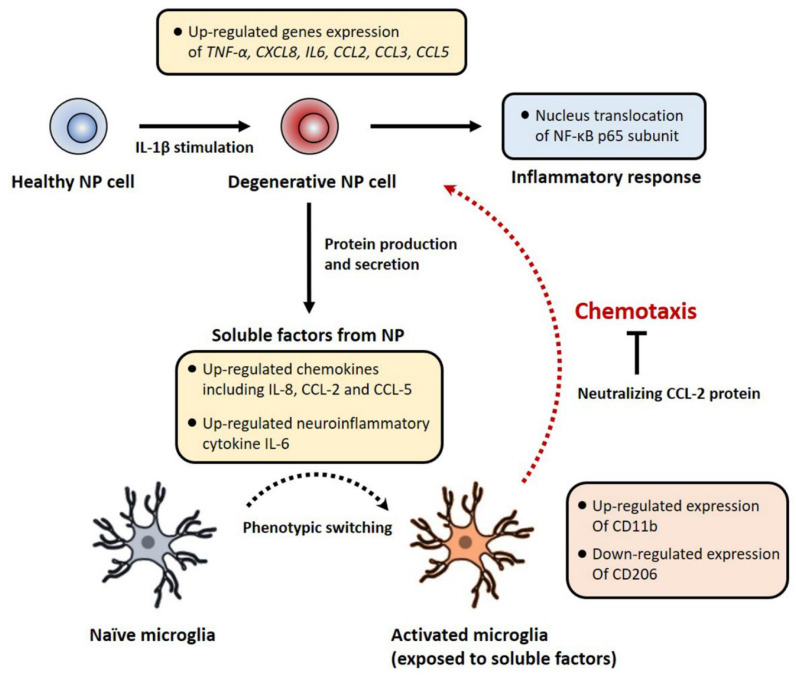
A schematic summary of the degenerative NP in vitro model and the effects of soluble factors derived from human NP cells on microglia’s chemotaxis and activation. In this study, IL-1β stimulation activates the NF-κB signaling pathway on human NP cells, which controls the expression of inflammatory and chemotactic genes encoding including *TNF-α, IL6, CXCL8, CCL2, CCL3* and *CCL5* via nucleus translocation of NF-κB p65 subunit. IL-1β-stimulated human NP cells show the protein production of IL-6, IL-8, CCL-2, and CCL-5. Conditioned medium containing these factors induces the recruitment and activation of microglia. Furthermore, neutralizing CCL-2 inhibits this chemotaxis effect in our microfluidic platform. Together, this pathomechanism can provide novel therapeutic target molecules for microglia-mediated neuroinflammation with LBP in IVD disease.

Our data demonstrated that soluble factors derived from degenerative human NP cells induced by IL-1β stimulation serve as recruiting signals during the recruitment of microglia. In particular, the chemoattractant CCL-2 directly caused microglial migration in response to soluble factors from degenerative human NP cells (Figure 6).

Evidence from the literature shows that several pro-inflammatory cytokines, such as IL-1β or TNF-α, are strongly correlated with IVD degeneration [2,9,20,21,22,23]. During IVD degeneration, these cytokines induce nerve growth factor (NGF) and vascular endothelial growth factor (VEGF) in human NP cells, which can result in neuronal ingrowth and angiogenesis in the central IVD region [15,24,25]. Moreover, these cytokines stimulate IVD cells to produce chemotactic factors that promote the recruitment of macrophages and monocytes. In fact, several chemokines, including CCL-2, -3, -4, -5, IL-6, and IL-8, were upregulated in both degenerated and herniated IVD tissues [2,26,27,28]. Moreover, IVD cells stimulated with IL-1β in vitro showed increased production of many of these cytokines [2,9,20,21,23]. In concert with other cytokine-responsive mediators, the expression of these factors is regulated through MAPK and p65-dependent NF-κB signalling pathways [22,29]. Similar to these studies, our results show that IL-1β stimulation of human NP cells induced the preferential distribution of NF-κB p65 protein into the nucleus rather than the cytoplasm, where it is associated with an inflammatory response by acting as a transcriptional factor. Furthermore, IL-1β-stimulated NP cells exhibited gene and protein expression of IL-6, IL-8, CCL-2, CCL-3, CCL-5, and TNF-α in contrast to those cultured in the naïve medium.

It is widely recognized that some of the inflammatory cytokines and chemokines during IVD degeneration are classically associated with immune cells, including macrophages, neutrophils, and microglia [2,15,30]. Some studies have shown the presence of immune cells, including activated T cells, B cells, macrophages, and natural killer (NK) cells, in degenerated and herniated IVDs. A study demonstrated that activated microglial cells may potentially concur with inflammation, secretion of pro-inflammatory mediators, and irritation of nerve fibers innervating the IVD [15,31]. This consideration leads to the hypothesis that human IVD cells may not be the sole contributors to the noxious and accelerating inflammatory responses found in degenerating IVD. Moreover, while the role of macrophage-mediated inflammation in IVD degeneration has been well demonstrated [32,33,34], little is known about how neuroinflammation, which can be mediated by microglia, is initiated and maintained in LBP.

First, in the probable mechanism of neuroinflammation initiation, diverse inflammatory responses are generally initiated by infiltration of immune cells, such as microglia, through invasive vascular structures. Indeed, most of the currently available studies support a positive correlation between increased innervation of the vascular/neuronal structure, discogenic pain, and inflammatory response [25,34,35]. Moreover, several studies have demonstrated that the degenerative IVD microenvironment includes infiltration and phenotypic switching of resident inflammatory-like cells, such as CD11b+, CD 68+, and CD4+ cells [2,32,36,37,38]. Consequently, invasive and activated microglia can interact with human NP cells in the inner IVD. Thus, it is crucial to explore the motility and phenotypic changes in microglia, as well as the factors that contribute to this phenomenon during IVD degeneration.

In this study, our microfluidic platform was employed as a single-cultured platform, which was described previously [17], to study human microglia chemotaxis in response to soluble factors from IL-1β-stimulated human NP cells. This chemotaxis platform was particularly advantageous to study microglia as we could separate only activated microglia in response to disease-associated soluble factors from heterogeneous populations involving spontaneously activated microglia and not responsible microglia. After the microglia separation, we could observe phenotypic changes in activated microglia using CD markers, such as CD11b and CD206, markers for M1/M2 immune cells, respectively. In addition, our results show that the degenerative microenvironment of human NP cells is able to induce the migration and activation of microglia through CCLs, particularly CCL-2, and inflammatory mediators, including IL-6 and IL-8, which are believed to be essential for the induction of IVD degeneration followed by neuropathic pain. We envision that our findings would contribute to the development of promising therapeutics preventing neuropathic pain. In addition, our IVD models developed in this study could be employed to test the new medicines for preventing or curing IVD-driven low back pain.

## 4. Materials and Methods

### 4.1. Isolation and Culture of Human NP Cells and Production of Conditioned Medium

Human NP cells were isolated from the disc tissues of twelve consenting patients (mean age ± SD = 54.87 ± 2.21; female:male = 4:8; Pfirrmann grade II–III) during surgical procedures for degenerative spinal disease in accordance with hospital regulations. The regulations and experimental protocols were approved by the Institutional Review Board of Korea University Hospital (KUGH17208-001), and written informed consent was obtained from all patients. Human IVD specimens were placed in Ham’s F-12 medium (F-12; Gibco-BRL, Gaithersburg, MD, USA) supplemented with 5% fetal bovine serum (FBS; Gibco-BRL, Gaithersburg, MD, USA) and 1% penicillin/streptomycin (P/S; Gibco-BRL, Gaithersburg, MD, USA). The disc tissues were washed three times using Hank’s balanced salt solution (HBSS; Gibco-BRL, Gaithersburg, MD, USA) containing 1% P/S, 5% FBS, and 0.2% Pronase (Calbiochem, La, Jolla, CA, USA), followed by incubation in a medium containing 0.025% collagenase for 24 h. Cells were filtered using a sterile nylon mesh (70-μm pore size) to remove tissue debris and isolate human NP cells. The supernatant was centrifuged at 676× *g* for 5 min and resuspended in the F-12 medium containing 10% FBS and 1% P/S. The isolated human NP cells were cultured in 75 cm^2^ cell culture flasks (VWR Scientific Products, Bridgeport, NJ, USA) in a humidified atmosphere with 5% CO_2_ at 37 °C.

To produce an experimental conditioned medium of NP cells, the cells were cultured in Dulbecco’s Modified Eagle Medium: Nutrient Mixture F-12 (DMEM/F12; Gibco-BRL, Gaithersburg, MD, USA) containing 1% FBS, 1% P/S, and with/without recombinant IL-1β (10 ng/mL, R&D Systems) for 48 h. In this study, the supernatant collected from NP cells is referred to as NP-conditioned medium without IL-1β (NPCM) and NP-conditioned medium with IL-1β (NPIL), respectively. The cells and supernatants were collected and stored at -80 °C for further experiments, including quantitative real-time polymerase chain reaction (qRT-PCR) and enzyme-linked immunosorbent assay (ELISA) analyses. Human NP cells were used at passage two.

### 4.2. Microglial Cell Culture

Human immortalized microglial cells (SV40) were purchased from Applied Biological Materials (ABM, Richmond, Canada) and cultured in the proprietary medium for 1–2 weeks according to the manufacturer’s instructions. The cells were cultured in Prigrow III medium (ABM; Richmond, CAN) supplemented with 10% FBS. Microglial cells at 80% confluence were removed from the culture flasks using 0.05% trypsin/EDTA (Gibco-BRL, Gaithersburg, MD, USA) and resuspended in the culture medium supplemented with 2% FBS. Prior to the experiment, the cell membrane was labeled with a red fluorescent dye (PKH26PCL, Sigma-Aldrich, St. Louis, MO, USA). Stained microglial cells in the culture medium at 5 × 10^4^ cells/mL were injected into the side wells of the microfluidic platform. The loaded cells were incubated in a humidified atmosphere with 5% CO_2_ at 37 °C for at least two days.

### 4.3. Fabrication Microfluidic Chemotaxis Platform

SU-8 photoresists were patterned using standard negative photolithography on a 4-inch silicon wafer to create a master mold for microchannels 20 μm in height, with each compartment 100 μm in height. A mixture of a base and curing agent in a 9:1 ratio by weight (SYLGARD 184 A/B, Dow Corning, Midland, MI, USA) was poured onto the master mold and cured at 70 °C in a dry oven for at least 2 h. The cured polydimethylsiloxane (PDMS) was detached from the mold and punched into the reservoirs. The arrayed holes were laser-cut (Zing 24, Epilog Laser, Golden, CO, USA) into a thin PDMS membrane with a thickness of 250 mm (HT 6240, Bisco Silicones, Elk Grove, IL, USA) and an acrylic plate with a thickness of 6 mm. The machined membrane and plate were glued together using uncured PDMS and incubated overnight at 80 °C.

### 4.4. Migration Test

We employed our chemotactic chip to test microglia response toward soluble factors in the ILNP. The microfluidic chemotaxis platform was composed of a central compartment (100 μm in height and 3 mm in diameter) and an angular compartment (100 μm in height and 3 mm in diameter), connected through microchannels (20 × 50 × 500 μm^3^ in height, width, and length, respectively). The microchannels were designed to be smaller than non-activated microglia, so that recruited only stimulated microglia in response to chemical gradients of soluble factors formed in the channels, as observed previously [17]. Other details of the chip design were described in our previous study. Before the experiment, each chamber in the platform was coated with 1.0 mg/mL poly-L-lysine (PLL; Sigma-Aldrich, St. Louis, MO, USA) to enhance cellular adhesion. Afterwards, microglial cells in the culture media supplemented with 2% FBS were plated into the annular compartment at an approximate density of 5 × 10^4^ cells/mL (in 100 µL of plating medium) per device. To avoid cell migration driven by hydrostatic pressure due to different levels in height between the two compartments, we also supplied same amount of plating medium (100 µL) in the central compartment. Upon the completion of microglia adhesion, we exchanged the central chamber medium into the conditioned medium (100 µL) collected by NP cells to verify the chemotaxis effect of potential contributing factors from IL-1β-stimulated NP cells on the microglial cells. To compare the effects of soluble factors from NP on microglial recruitment, we evaluated the fraction of microglial cells recruited to the central compartment after loading the conditioned medium. To further demonstrate CCL2 as a key chemokine in NPIL to recruit microglia, we added NPCM or NPIL supplemented with blockage antibody for CCL2 (ab CCL2, 1 µg/mL) and monitored microglia migration.

### 4.5. Quantitative Real-Time Polymerase Chain Reaction (qRT-PCR)

The mRNA of human NP cells was isolated using TRIzol reagent (Invitrogen, Waltham, MA, USA); mRNA was purified using an RNA miniprep kit (Zymo Research, Irvine, CA, USA), and cDNA was synthesized using a cDNA synthesis kit (Life Technologies, Carlsbad, CA, USA). Upon confirming the quantity and quality of cDNA, qRT-PCR analysis was performed on TNF-α, IL6, CXCL8, CCL2, CCL3, and CCL5 using SYBR PCR Master Mix (Applied Biosystems, Foster city, CA, USA). The mRNA expression was analyzed using the 2^−∆∆Ct^ method, and values are presented as the mean fold change relative to the normalized levels of the housekeeping gene glyceraldehyde 3-phosphate dehydrogenase (GAPDH).

### 4.6. Enzyme-Linked Immunosorbent Assay (ELISA)

The quantity of protein produced from human NP cells was analyzed using a commercially available ELISA kit (R&D Systems, Minneapolis, MN, USA) according to the manufacturer’s instructions. Accordingly, we analyzed the protein production of CCL-2, CCL-3, CCL-5, IL-6, and IL-8 in the supernatants.

### 4.7. Immunofluorescence Staining

Microglial cells on the microfluidic chip were fixed with 4% paraformaldehyde and permeabilized with 0.2% Triton X-100 in phosphate-buffered saline (PBS; Gibco-BRL, Gaithersburg, MD, USA) for 15 min at room temperature (RT). Blocking was performed using 3% bovine serum albumin (BSA; Millipore, MN, USA) in PBS (1% *w*/*v*) for approximately 2 h at RT. Subsequently, the cells were incubated with CD11b (Millipore, MN, USA) and CD206 (Novus biologicals, CO, USA) primary antibodies for 2 h at 37 °C at a dilution ratio of 1:100 and washed with PBS containing 1% BSA. Thereafter, the cells were incubated with Alexa 555 secondary antibody (1:200; Invitorgen, Waltham, MA, USA) for 2 h at 37 °C. 

Human NP cells were treated with recombinant IL-1β for 48 h and fixed and blocked with proprietary reagents. Anti-NF-κB p65 mouse monoclonal antibody (Santa Cruz, Dallas, TX, USA) was used to detect NF-κB p65 protein. Goat anti-mouse Alexa 555 secondary antibodies (Invitrogen, Waltham, MA, USA) and 5% BSA were used for secondary incubation in PBS for 1 h at room temperature. After staining with 4′,6-diamidino-2-phenylindole (DAPI) for 10 min, fluorescence images were acquired using a confocal microscope (Zeiss, LSM 900).

### 4.8. Cell Cytotoxicity and Lactate Dehydrogenase Assay

The release of lactate dehydrogenase (LDH) was measured according to the manufacturer’s instructions. After microglial cells were exposed to NPCM or NPIL, the exposure medium was collected to quantify the release of LDH. Viability was calculated with respect to the control (naïve microglial cells). If the microglial cells were damaged by a conditioned medium, they would tend to have an increased LDH production.

### 4.9. Statistical Analysis

All experimental data are expressed as mean ± standard error of three individual experiments and four independent groups. Statistical significance was calculated using one-way analysis of variance (ANOVA) with a Bonferroni calibrated post hoc test; statistical significance was set at *p* < 0.05. All the statistical analyses were performed using GraphPad Prism 9.0 (GraphPad Software, La Jolla, CA, USA).

## 5. Conclusions

Herein, we evaluated the chemotaxis effects on human microglial cells using endogenous cues derived from human NP cells, which are expected to occur during microglial-mediated inflammation in IVD tissues. Our results provide striking evidence for a direct link between degenerative NP cells and the activation of microglial cells that exacerbate the degenerative microenvironment within the deeper IVD and peridiscal space during the herniation of NP tissue. The development of anti-inflammatory therapeutic strategies for IVD degeneration may be aided by a better understanding of microglial cell responses to endogenous cues produced by human IVD. Furthermore, a newly developed IVD chip can be applied in studies of cell-to-cell interactions during various diseases, especially in terms of live, systematic monitoring.

## Data Availability

The datasets generated and/or analyzed during the current study are available from the corresponding author upon reasonable request.
